# Increased phosphorylation of p70 S6 kinase is associated with HPV16 infection in cervical cancer and esophageal cancer

**DOI:** 10.1038/sj.bjc.6603838

**Published:** 2007-07-10

**Authors:** Y Zhou, Y Pan, S Zhang, X Shi, T Ning, Y Ke

**Affiliations:** 1 Beijing Institute for Cancer Research, School of Oncology, Peking University No. 52, Fucheng Rd, Hai Dian District, Beijing 100036, PR China; 2 Department of Surgery, Anyang Cancer Hospital, Anyang City, Henan Province 455000, PR China; 3 Department of Cell Biology, Health Science Center, Peking University No. 38, Xueyuan Rd, Hai Dian District, Beijing 100083, PR China; 4 Cancer Research Center, Health Science Center, Peking University No. 38, Xueyuan Rd, Hai Dian District, Beijing 100083, PR China

**Keywords:** S6 kinase, HPV, Akt, cervical cancer, esophageal cancer

## Abstract

HPV16 E6 interacts with and degrades tumour suppressor protein TSC2 leading to the phosphorylation of p70 S6 kinase. We studied the association of S6 kinase phosphorylation and HPV16 infection in cervical cancer and esophageal cancer. Immunohistochemistry was used to assess phosphorylated S6 kinase (Thr 389) and phosphorylated S6 (Ser235/236) in 140 cervical cancer and 161 esophageal cancer specimens. Immunohistochemical staining for pS6 kinase and pS6 was significantly more frequent in the HPV16-infected cervical cancer specimens than the HPV16-negative specimens. In contrast, the expression of S6 kinase was similar in both HPV16-positive and -negative samples. The phosphorylation of Akt, the key regulator of S6 kinase, was also detected. Our analysis showed that Akt phosphorylation was unaffected by HPV16 infection. These results together with our previous study suggest that HPV16 modifies S6 kinase activation via mechanism, which activates S6 kinase downstream of Akt function.

Human papillomavirus (HPV) is a causal agent of cervical cancer and a risk factor for the other epithelial cancer development including esophageal squamous cell carcinomas (zur Hausen, 1999; Bosch *et al*, 2002; Kari Syrjanen, 2006). Two viral proteins, E6 and E7, are expressed in nearly all cervical cancers and are both necessary and sufficient for immortalisation of primary human keratinocytes *in vitro* (Hawley-Nelson *et al*, 1989; Munger *et al*, 1989; Hudson *et al*, 1990). The inactivation of tumour suppressor proteins, p53 and Rb by E6 and E7, respectively, plays the major role in HPV-induced tumourigenesis (Dyson *et al*, 1989; Munger *et al*, 1989; Scheffner *et al*, 1990; Werness *et al*, 1990; Heck *et al*, 1992). In addition, a growing number of proteins involved in the broad spectrum of cellular functions have been found to be interfered by E6 or E7. We have previously identified tumour suppressor protein TSC2 as the novel target of HPV16 E6. HPV16 E6 interacts with TSC2 and causes the proteasome-mediated degradation of TSC2 resulting in the phosphorylation of S6 kinase even in the absence of insulin (Lu *et al*, 2004).

Recent studies have revealed that TSC2 plays an important role in the cell growth and proliferation pathway, in which TSC2 forms a functional complex with TSC1 and negatively regulates both growth factor and nutrient-dependent activation of mTOR signalling to its downstream targets S6 kinase and 4EBP1 (Gao *et al*, 2002; Manning and Cantley, 2003; Saucedo *et al*, 2003; Stocker *et al*, 2003; Hay and Sonenberg, 2004). The negative regulatory role of the complex is achieved by TSC2 GTPase-activating protein activity towards Rheb. Abrogation of TSC2 function by HPV16 E6 may therefore contribute to HPV16-induced proliferation and tumourigenesis.

In this study, we evaluate the phosphorylation of S6 kinase using immunochemical staining in cervical cancer and esophageal cancer tissues and have correlated pS6K staining with HPV16 infection in the patients studied.

## MATERIALS AND METHODS

### Patient selection

One hundred and forty cervical cancers diagnosed between 2000 and 2004, and 161 esophageal cancers diagnosed in 2004 were identified from Anyang Cancer Hospital, China. Tumour material consisted of formalinfixed, paraffin-embedded tumour blocks. Tumours were histologically typed and graded.

### Detection of HPV16 infection

DNA was extracted from the paraffin sections as described (Nindl *et al*, 2004). For HPV16 DNA detection, E7 was amplified with the forward primer 5′-GATGAAATAGATGGTCCAGC-3′ and the reverse primer 5′-GCTTTGTACGCACAACCGAAGC-3′ as described (Walboomers *et al*, 1999). In PCR reaction, *β*-actin PCR analysis was used to validate the DNA quality. Mouse liver tissue was used in the sample process and PCR analysis to monitor the contamination. PCR mixtures contained 10 mM Tris–HCl (pH 8.3), 50 mM KCl, 3.5 mM MgCl_2_, 0.01% gelatin, 200 pmol each primer, 4 U *Taq* DNA polymerase (Promega, Madison, WI, USA) and 100 ng DNA. The cycling conditions were 94°C for 40 s, 57°C for 40 s and 72°C for 40 s for 40 cycles.

### Immunohistochemistry

Sections were deparaffinised in xylene, washed in graded ethanol and followed in phosphate-buffered saline (PBS). The endogenous peroxidase activity was blocked with PBS containing 3% hydrogen dioxide. Samples were then pretreated with target retrieval citrate buffer in a microwave for 15 min and washed three times with PBS.

**Figure 1:**
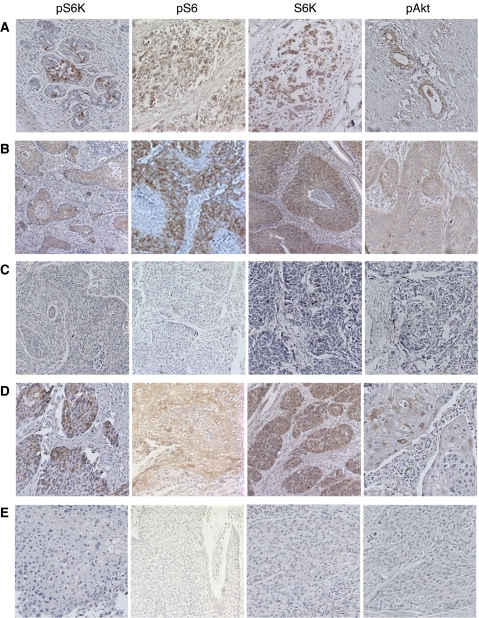
Immunohistochemistry staining of pS6K, pS6, S6K and pAKT in cervical cancer and esophageal cancer tissues. (**A**) Positive control of pS6K, pS6, S6K and pAKT in breast cancer (pS6K, pS6, S6K) and prostate cancer (pAKT) tissues. (**B**) Positive staining of pS6K, pS6, S6K and pAKT in cervical cancer tissues. (**C**) Negative staining of pS6K, pS6, S6K and pAKT in cervical cancer tissues. (**D**) Positive staining of pS6K, pS6, S6K and pAKT in esophageal cancer tissues. (**E**) Negative staining of pS6K, pS6, S6K and pAKT in esophageal cancer tissues.

The nonspecific binding was blocked by incubation in PBS containing 10% goat serum for 1 h. Slides were stained with IHC-specific phosphorylated Thr 389 S6K antibody (1 : 500 dilution, Cell Signalling Technologies Inc., Beverly, MA, USA), S6K antibody (1 : 500 dilution, Santa Cruz Biotechnology, Santa Cruz, CA, USA), phosphorylated Ser 235/236 S6 antibody (1 : 100 dilution, Cell Signalling Technologies Inc.) and phosphorylated Ser 473 Akt antibody (1 : 200 dilution, Cell Signalling Technologies Inc.) at 4°C overnight. The biotinylated secondary antibody and HRP-labelled streptavidin were then added and incubated at 37°C for 30 min. The signal was developed in DAB–H_2_O_2_ solution. The slides were counterstained with hemotaxylin, and then examined by light microscopy. The representative slides stained by different antibodies are shown in Figure 1. The staining was scored on a scale from 0 to IV as follows: 0, less than 5% cells were stained; I, 5–25% cells were stained; II, 25–50% cells were stained; III, 50–75% cells were stained; and IV, more than 75% cells were stained. Scores I–IV were classified as positive, while score 0 was negative.

### Statistical analysis

The *χ*^2^-test was used to investigate the independence of categorical variables. The *P*-value reported was two sided, and values of *P*<0.05 were considered significant. Statistical analysis was performed and statistical calculations were carried out using SAS 8.0.

## RESULTS

Patient characteristics are listed in Table 1. Since we have found that TSC2 specifically binds to HPV16 E6 and leads to the elevated S6 kinase phosphorylation independent of extra cellular stimuli, to examine if upregulation of active S6 kinase occurs *in vivo* as a result of HPV16 infection, we determined phosphorylated S6 kinase in cervical cancer and esophageal cancer with or without HPV16 infection. The presence of HPV16 was detected by amplification of HPV16 E7 gene fragment. Among 140 cervical cancer specimens, 102 were HPV16 positive (72.9%). In case of esophageal cancer, 97 out of 160 (60.2%) were HPV16 positive.

The phosphorylation of p70 S6 kinase was detected using immunohistochemical stain with antibody specifically against phosphorylated S6 kinase (Thr 389). The degree of immune staining was scored as described in Materials and Methods. In cervical cancer, 43 (42.2%) HPV16-positive specimens were scored pS6K staining positive I and above, while 3 (7.9%) HPV16-negative specimens were stained with the same degree (*P*=0.000). Thirty (29.4%) HPV16 positives and 1 (2.6%) HPV16 negatives were stained as degree II and above (*P*<0.002) (Table 2).

In order to confirm the effect of HPV16 on the activation of S6 kinase, we detected the phosphorylation of S6 using anti-phospho-S6 antibody (Ser235/236). Seventy-one (69.6%) of HPV16-positive specimens were scored degree I and above, and 51 (50%) of HPV16 positives were degree II and above. In contrast, 12 (31.6%) of HPV16 and 9 (23.7%) of HPV16 negatives were found in the corresponding groups (*P*=0.000 and *P*<0.009, respectively).

In esophageal cancer, 26 (26.8%) HPV16-positive specimens were pS6K positive with degree I and above staining, while 5 (7.8%) of HPV16 negatives were pS6K positive with corresponding degree (*P*<0.005). No significant difference was found between HPV16 positives and negatives with degree II and above pS6K-positive staining. No significance of pS6 staining at any degree was found between HPV16 positives and negatives (Table 3).

In order to exclude the possibility that elevated S6 kinase, phosphorylation was caused by the consequence of increased S6 kinase expression, the specimens were stained with anti-S6 kinase antibody. A 78.4% of HPV16-positive and 78.9% of HV16-negative cervical cancers were S6K positive with degree I and above (*P*=1.000). In esophageal cancers, 61.9% of HPV16 positives and 56.3% of HPV16 negatives were S6K positive with degree I and above (*P*=0.586).

The serine/threonine kinase Akt plays a central role upstream mTOR and S6 kinase in PI3Kinase pathway. The activity of AKT is upregulated in many human cancers leading to S6 kinase phosphorylation (Brazil *et al*, 2002; Altomare and Testa, 2005). We next tested the phosphorylation of Akt in HPV16-infected cancer specimens. An antibody specifically against phosphorylated Akt (Ser 473) was used for immunohistochemical stain. In cervical cancer group, 9.8% HPV16 positives and 13.2% HPV negatives were pAkt positive with degree I and above (*P*=0.792). In esophageal cancer group, 6.2% HPV16 positives and 4.7% HPV16 negatives were pAkt positive with degree I and above (*P*=0.957).

The representative examples of pS6K, pS6, S6K and pAkt staining are shown in Figure 1. The brownish signals represent the positive staining. The phosphorylated S6K and S6 were found mainly in the cytoplasm, while S6K was in both cytoplasm and nucleus. The phosphorylated Akt was mainly in the cytoplasm. The positive controls used were a breast cancer specimen for S6K, pS6 and pS6K, and a prostate cancer specimen for pAkt.

## DISCUSSION

Human papillomaviruses play an etiological role in the development of cervical cancer and several other cancers. Oncogenic HPV E6 and E7 interrupt the host cellular function and therefore take the principal contribution for HPV-induced tumourigenesis. We have previously reported that HPV16 E6 binds to tumour suppressor protein TSC2 and inferences with its suppressor function in insulin-induced cell growth and proliferation-signalling pathway leading to activate S6 kinase downstream of Akt activation (Lu *et al*, 2004). In the present study we demonstrate that S6 kinase phosphorylation was significantly increased in HPV16-infected cervical cancer. In parallel, the phosphorylation of S6 augmented. In HPV16-infected esophageal cancer specimens, the alteration of S6 kinase phosphorylation was not as significant as in cervical cancer specimens. In particular, there was no difference of S6 phosphorylation between HPV16-positive and -negative specimens. The discrepancy between cervical cancer and esophageal cancer might be resulted from much lower detectable level of pS6 kinase and pS6 detected in esophageal cancer specimens. Alternatively, the expression of HPV16 E6 may differ in those two group specimens and subsequently affect the activation of S6 kinase differently. We also showed that increased phosphorylation of S6 kinase was resulted from neither elevated S6 kinase expression nor Akt activation in specimens with HPV16 infection.

TSC2 is a key negative regulator of mTOR signalling pathway, in which TSC2 acts as a GTPase-activating protein towards Rheb, thereby inhibiting mTOR. Phosphorylation of TSC2 influences its activity within the complex with TSC1. At least three kinases, Akt, RSK1 and MAPK, are involved in TSC2 phosphorylation (Jozwiak, 2006). Several studies reported that HPV proteins target the components of PI3 kinase and Akt-mediated cell growth pathway. For instance, HPV E6 proteins interact with the membrane-associated guanylate kinase homologues (MAGUKs) MAGI-2 and MAGI-3 for degradation (Thomas *et al*, 2002). MAGI-2 and MAGI-3 have been reported to be associated with PTEN (Wu *et al*, 2000), the other negative regulator of the insulin-induced growth pathway, and enhance the ability of PTEN to suppress Akt activation. The degradation of MAGI-2 and MAGI-3 by E6 may, therefore, result in the abolishment of PTEN function resulting in the activation of Akt-mediated cell growth. In addition, HPV-16 E7 upregulates AKT activity in both Rb-dependent and -independent manner (Westbrook *et al*, 2002; Pim *et al*, 2005; Menges *et al*, 2006). Taken together, these results highlight the importance of interference with the PI3 kinase/Akt-mediated signalling pathway in HPV-induced tumourigenesis. In the current study, we did not observe enhanced Akt phosphorylation in HPV16-positive specimens. This might be caused by that immunohistochemistry is not sensitive enough to detect Akt activation in HPV-infected samples. However, it is also possible that HPV proteins direct target components of this pathway downstream of Akt, or even independent of this pathway in certain condition. Therefore, the activation of Akt might remain unchanged. For instance, our previous study showed that expression of HPV16 E6 promoted the degradation of TSC2 and phosphorylation of S6 kinase and S6 in the absence of insulin stimulation, while phosphorlation of Akt was unchanged in the presence and absence of HPV16 E6 (Lu *et al*, 2004). The results presented here support our previous study.

In addition, HPV proteins also activate MAPK pathway. HPV16 E6 increases the phosphorylation of MAPK and MER (Chakrabarti *et al*, 2004). HPV16 E5 promotes the activation of EGFR and thus enhances the RAS/RAF/MAPK signalling cascade (Kim *et al*, 2006). Further investigation on why and how HPV oncoproteins preferentially target the different components in this pathway will be of great help for understanding HPV-induced tumourigenesis.

Recent studies also found that amplification of the PIK3CA gene accompanied by serine 473 phosphorylation of AKT is common in cervical cancers and it seems to be an independent event to HPV infection (Ma *et al*, 2000; Zhang *et al*, 2002; Bertelsen *et al*, 2006). Under our current condition, only 9.8% of cervical cancers and 6.2% of esophageal cancers were pAkt positive. This discrepancy is unlikely due to a technical issue since we have repeated the detection using antibodies from different batches and a positive control from the manufacture was always used to monitor the sensitivity. One possible explanation is that PIK3CA gene amplification is corresponding to the progression of disease. In our analysis, tumours were not classified by stages. In addition, Kim *et al* (2006) reported that Akt phosphorylation contributes to radioresistance in cervical cancer. A more detailed analysis is required to define the Akt phosphorylation-related disease stage and progression.

## Figures and Tables

**Table 1:** Patient characteristics

	**Cervical cancer (*N*=140)**	**ESCC (*N*=161)**
*Age (year)*		
Median (range)	48 (21–80)	58 (36–77)
		
*Cell type* (%)		
Squamous cell	140 (100)	161 (100)
		
*HPV status* (%)		
HPV 16+	102 (72.9)	97 (60.2)
HPV 16−	38 (27.1)	64 (39.8)
		
*Histologic grade*		
High-differentiated tumour	7	11
Mild-differentiated tumour	71	102
Low-differentiated tumour	47	48
Undefined cancer	15	0

**Table 2:** Immunohistochemical analysis for p-S6K, p-S6, S6K and p-AKT in cervical cancer tissues

			**IHC score**								
	**Group**	**Total number**	**IV**	**III**	**II**	**I**	**0**	**⩾I (%)**	***P***	**⩾II (%)**	***P***
p-S6K	HPV16+	102	10	5	15	13	59	43 (42.2)		30 (29.4)	
	HPV16−	38	1	0	0	2	35	3 (7.9)	0.000	1 (2.6)	0.002
p-S6	HPV16+	102	18	13	20	20	31	71 (69.6)		51 (50.0)	
	HPV16−	38	4	3	2	3	26	12 (31.6)	0.000	9 (23.7)	0.009
S6K	HPV16+	102	41	11	12	16	22	80 (78.4)		64 (62.7)	
	HPV16−	38	12	8	4	6	8	30 (78.9)	1.000	24 (63.2)	1.000
p-Akt	HPV16+	102	2	1	3	4	92	10 (9.8)		6 (5.9)	
	HPV16−	38	0	0	3	2	33	5 (13.2)	0.792	3 (7.9)	0.965

**Table 3:** Immunohistochemical analysis for p-S6K, p-S6, S6K and p-AKT in ESCC tissues

			**IHC score**								
	**Group**	**Total number**	**IV**	**III**	**II**	**I**	**0**	**⩾1 (%)**	***P***	**⩾2 (%)**	***P***
p-S6K	HPV16+	97	6	5	4	11	71	26 (26.8)		15 (15.5)	
	HPV16−	64	1	1	2	1	59	5 (7.8)	0.005	4 (16.3)	0.128
p-S6	HPV16+	97	2	1	5	15	74	23 (23.7)		8 (8.2)	
	HPV16−	64	0	0	2	7	55	9 (14.1)	0.194	2 (3.1)	0.325
S6K	HPV16+	97	9	12	15	24	37	60 (61.9)		36 (37.1)	
	HPV16−	64	6	10	7	13	28	36 (56.3)	0.586	23 (35.9)	1.000
p-Akt	HPV16+	97	0	0	0	6	91	6 (6.2)		0	
	HPV16−	64	0	0	0	3	61	3 (4.7)	0.957	0	1.000
